# From metabolic syndrome to survival: a prognostic model for PDAC incorporating insulin resistance markers and pathological variables

**DOI:** 10.3389/fendo.2026.1942873

**Published:** 2026-09-03

**Authors:** Tao Zhang, Xue Li, Yingli Guo, Junsong Zeng, Maosen Xu, Tingting Wang

**Affiliations:** 1 Department of Biotherapy, Cancer Center and State Key Laboratory of Biotherapy, West China Hospital, Sichuan University, Chengdu, China; 2 State Key Laboratory of Biotherapy and Cancer Center, West China Hospital, Sichuan University, Chengdu, China; 3 Department of Psychiatry, Fundamental and Clinical Research on Mental Disorders Key Laboratory of Luzhou, Affiliated Hospital of Southwest Medical University, Luzhou, China

**Keywords:** insulin resistance, metabolic syndrome, overall survival, pancreatic ductal adenocarcinoma, prognostic model, triglyceride-glucose index

## Abstract

**Background:**

Metabolic disorders are increasingly recognized as key players in pancreatic ductal adenocarcinoma, yet their prognostic value after surgery remains unclear. Simple and reliable markers such as the triglyceride-glucose (TyG) index offer a practical way to capture insulin resistance and related metabolic disturbances.

**Method:**

We retrospectively enrolled 506 patients who underwent curative resection for PDAC. Based on preoperative clinical and postoperative pathological data, we developed three Cox regression models to predict 1-year and 3-year overall survival: a clinical model, a pathological model, and a combined model that integrated both. We evaluated discrimination, calibration, decision curves, and risk stratification, with particular focus on the TyG index and other metabolic variables.

**Result:**

The combined model outperformed the others, achieving a validation C-index of 0.721 and AUCs of 0.821 (1-year). The TyG index consistently emerged as the strongest independent predictor across all models (HR up to 1.50). Subgroup analysis by TNM stage revealed that the prognostic impact of the TyG index, body mass index, and albumin varied substantially with disease stage. Decision curve analysis confirmed the combined model’s clinical utility, especially when more aggressive intervention is considered.

**Conclusion:**

We developed and validated three prognostic models for resected PDAC based on routine clinical and pathological data. The combined model, which integrates metabolic markers such as the TyG index with traditional pathological features, showed the best discrimination and clinical utility. Our results underscore the important role of metabolic dysregulation in postoperative survival. However, these models should serve as supportive tools to inform clinical decision-making rather than replace comprehensive patient assessment.

## Introduction

Pancreatic ductal adenocarcinoma (PDAC) accounts for over 90% of pancreatic malignancies and remains one of the most lethal solid tumors (1). The prognosis is poor because PDAC typically invades adjacent structures early and has already seeded distant micrometastases by the time of diagnosis. Although surgical resection offers the only potentially curative option, five-year survival after apparently successful surgery seldom reaches 30%, owing to frequent local recurrence and occult metastatic spread (2–4). Identifying patients at highest risk of early postoperative death is therefore critical for guiding decisions about adjuvant chemotherapy and surveillance intensity.

Over the past decade, substantial attention has shifted to the interplay between metabolic dysfunction and cancer. Components of the metabolic syndrome—central obesity, insulin resistance, and dyslipidemia—show consistent links to an increased incidence of several malignancies, including lung, breast, and pancreatic cancers (5–8). Because the pancreas serves a dual role as both an exocrine digestive gland and an endocrine metabolic regulator, systemic metabolic disturbances likely hold particular relevance for pancreatic carcinogenesis (9). Long-standing type 2 diabetes, for instance, roughly doubles the risk of developing PDAC, and many now regard it as an independent predisposing factor (10, 11). At the same time, PDAC itself frequently triggers new-onset diabetes by disrupting the organ’s ability to maintain glucose homeostasis (12–14). This bidirectional relationship implies that metabolic status may fuel tumor progression while also representing a consequence of the tumor—a self-reinforcing cycle.

Despite the recognized importance of metabolic disorders in PDAC development, clinical models for postoperative prognostication rarely incorporate metabolic biomarkers. One promising candidate is the triglyceride-glucose (TyG) index, a simple, low-cost, and reproducible surrogate for insulin resistance that has been validated across diverse populations (15, 16). The TyG index reflects systemic insulin sensitivity without needing direct insulin measurements. For routine clinical practice, the TyG index is more economical and easier to obtain. In patients with advanced pancreatic cancer, the TyG index has been linked to tumor progression (17, 18). We have not systematically explored the prognostic value of this index in resectable PDAC patients at an early stage.

Classical prognostic factors for pancreatic cancer include lymph node metastasis, tumor differentiation, resection margin status, and serum tumor biomarker levels (19–23). More recent studies have explored molecular markers such as KRAS mutations, microRNA signatures, and circulating tumor DNA (24–27). Radiomics-based models have also shown promise in predicting survival (28–30). In routine clinical practice, these approaches are difficult to implement widely because of their relatively high cost. In contrast, the clinical and pathological data collected during standard preoperative work-up and postoperative examination are readily available for building prognostic models.

In this study, we aim to develop and validate prediction models based on preoperative clinical and postoperative pathological information to predict overall survival (OS) in PDAC patients who undergo surgery. Our models rely only on routinely collected clinical and pathological data, with a special focus on the role of the TyG index in the metabolic-pancreatic cancer axis. We hope to provide easy-to-use prognostic tools that can support clinical decision-making for patients under various medical conditions.

## Method

### Study population

We retrospectively identified patients who underwent curative resection for PDAC between June 2016 and May 2023 at West China Hospital. Patients who had received preoperative anticancer therapy, had a concurrent malignancy, or had incomplete clinical records or pathological results were excluded.

### Data collection

Clinical and pathological data were retrieved from the medical records. For each laboratory parameter, the measurement closest to the date of surgery was selected. Clinical variables fell into three categories: demographics, medical history (smoking, hypertension, diabetes), and preoperative laboratory tests. Pathological information including tumor location and tumor length, defined as the longest lesion diameter, was obtained primarily from the surgical report. TNM stage was assigned based on postoperative pathology. For histological grading, reports describing the tumor as “moderately to poorly differentiated” were recorded as moderately differentiated; those described as “moderately to well differentiated” were recorded as well differentiated. All patients were followed until December 30, 2025. Survival time was calculated in months from the date of diagnosis until death or last follow-up.

### Data preprocessing

We first converted all categorical variables, including demographics, comorbidities, and tumor staging information, into factors. To minimize the impact of missing data while preserving sample size, we imputed missing values using the median for continuous variables and the mode for categorical ones. Variables with over 50% missing, along with redundant individual staging components (T, N, and overall stage), were excluded, leaving TNM stage as the primary pathological staging variable. After completing data cleaning, we randomly split the entire cohort into a training set and a validation set in a 7:3 ratio. A stratified sampling strategy based on survival status was applied with a fixed seed of 123 to ensure a balanced distribution of outcomes and full reproducibility. The training set served for all model development and variable selection, while the validation set was reserved strictly for performance evaluation.

### Model construction and variable selection

Our primary goal was to build and compare three models for predicting overall survival after surgery. We started by performing univariate Cox regression on all 25 clinical variables and the four pathological variables (tumor location, tumor length, TNM stage, and pathological grading). All variables with a suggestive association (p < 0.10) in the univariate analysis were carried forward to the multivariable stage. For the clinical and pathological models, we employed a backward stepwise selection procedure, removing variables with a p-value > 0.05 until only significant predictors remained. This approach was chosen to reduce model complexity while retaining potentially important covariates. The combined model was then constructed by fitting a multivariable Cox regression on the union of all variables selected in the final clinical and final pathological models, without further elimination.

### Risk threshold and nomogram

For each continuous predictor retained in the combined model, a univariate Cox regression was refitted to generate an individual risk score. The optimal threshold was determined by maximizing Youden’s index using the ROC curve, with 12-month mortality as the endpoint. Using the final variables of the combined model, we constructed a nomogram from the training set to estimate the probability of 1-year and 3-year survival. These two time points were selected to align with clinically relevant milestones in the postoperative course of resected PDAC: 12-month survival captures early outcomes and informs decisions regarding adjuvant therapy intensity and early surveillance, while 36-month survival approximates longer-term disease control and reflects the durability of initial treatment effects. Given that PDAC recurrence typically peaks within the first 2 years after resection, these horizons together provide a clinically meaningful framework for prognostic stratification.

### Discrimination and calibration

We assessed model performance through discrimination and calibration in both the training and validation sets. Discrimination was quantified by the C-index with 95% confidence intervals and by time-dependent AUC. Using the optimal risk threshold obtained from the training set, we further calculated accuracy, sensitivity, specificity, positive predictive value, and negative predictive value. Calibration was evaluated by grouping patients into ten equal-sized bins based on predicted survival probability at 12 months and 36 months. Within each bin, the mean predicted probability was compared against the observed probability estimated by the Kaplan-Meier method. A linear regression of observed on predicted probabilities yielded the calibration slope, intercept, and R-squared. Bootstrap resampling (200 replicates) provided 95% confidence intervals for the observed probabilities.

### Risk stratification, subgroup analysis and decision curve analysis

To explore the prognostic value of the risk scores, we stratified patients into high-risk and low-risk groups using the median risk score from the training set as a cutoff. Kaplan-Meier curves were generated for both training and validation cohorts. We further examined whether the risk classification remained informative across TNM stages by performing subgroup analyses, fitting a Cox model with risk group as the predictor in each stratum. Histograms of the three models’ risk scores were plotted from the training set. Decision curve analysis, based on the net benefit, was conducted at 1 and 3 years for both cohorts, with “treat-all” and “treat-none” included as references.

### Software and significance

The linear predictors of all three Cox models were extracted, and risk score formulas were expressed as risk score = exp(Σβi × Xi). All analyses were carried out using R (version 4.6.0). Statistical significance was set at a two-sided p-value below 0.05. The proportional hazards assumption was verified for all final models using Schoenfeld residual tests. For variables that showed evidence of time-varying effects, we included time-dependent interaction terms using a logarithmic time transformation.

## Results

### Baseline characteristics of the study population

**Figure 1:**
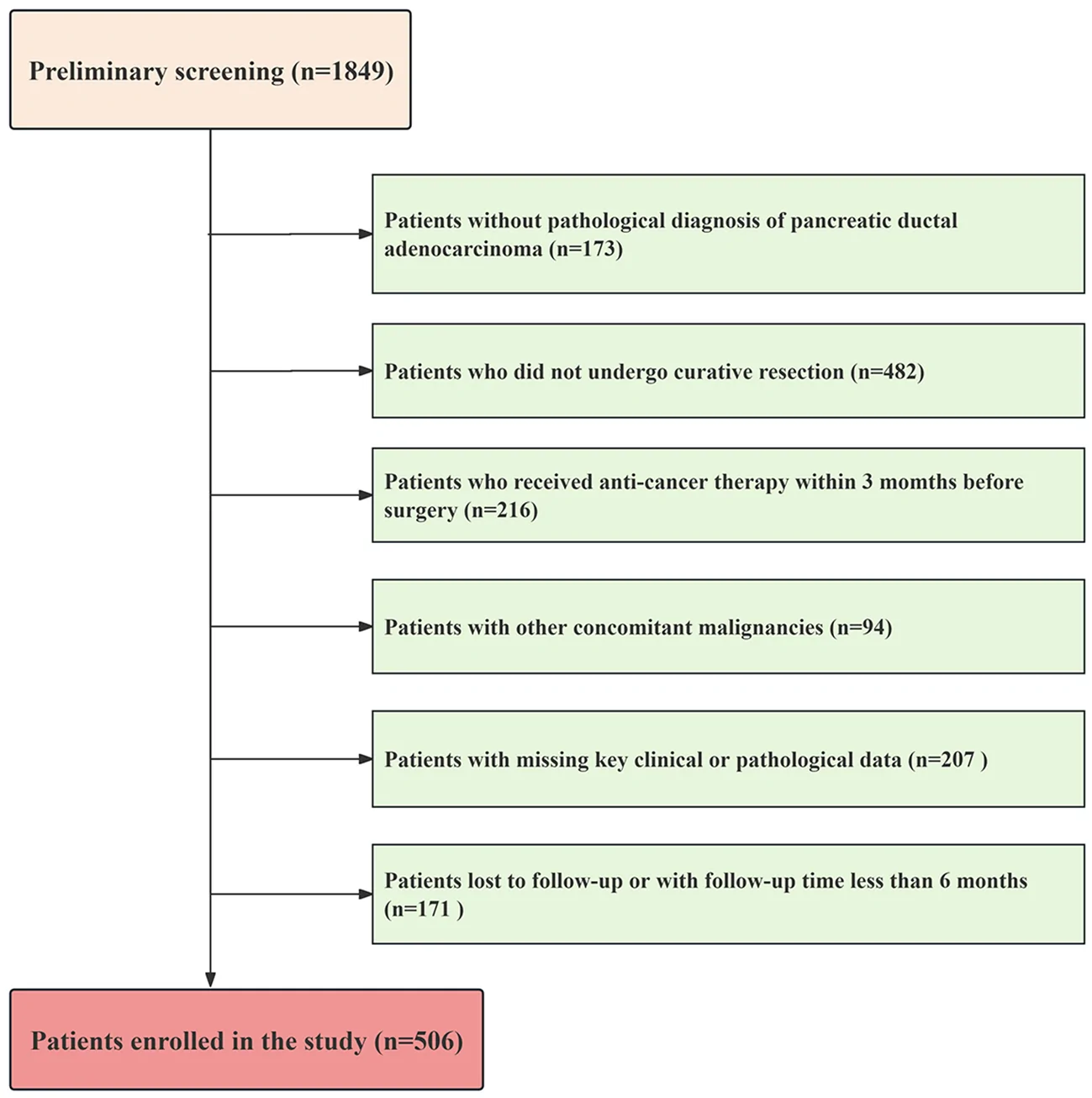
Flowchart of patient selection.

A total of 1849 patients were initially screened. Among them, 173 patients who did not have a pathological diagnosis of pancreatic ductal adenocarcinoma were excluded. We removed 482 patients who did not undergo curative resection and 216 patients who received anti-cancer therapy within three months before surgery. Another 94 patients had concomitant malignancies. We also excluded 207 patients with incomplete key clinical or pathological data and 171 patients who were lost to follow-up or had a follow-up time shorter than six months. The final cohort comprises 506 patients (**Figure 1**).

The 506 enrolled patients were randomly assigned to a training set (n = 355) and a validation set (n = 151). Their demographic and clinical profiles were broadly comparable. Men made up roughly 60% of both groups. Mean age ranged between 57 and 58 years. Smoking history, hypertension, and diabetes were each present in about one-third to two-fifths of patients. Tumors arose predominantly from the pancreatic head (about 68%) and averaged 4.1 cm in length. T2 and T3 stages accounted for over 80% of cases, and N1 disease accounted for roughly half. T2N1M0 and T3N1M0 were the most common TNM subgroups. Moderately differentiated tumors (G2) dominated at 68.7% and 72.8% in the training and validation sets, respectively. Mean fasting blood glucose exceeded the upper normal limit in both sets (6.89 and 6.75 mmol/L), and triglyceride levels averaged 2.2 mmol/L, yielding mean TyG indices above 9.2. CA19-9 levels were markedly elevated. Other laboratory parameters were largely comparable, though amylase and lipase were slightly higher in the validation set. **Table 1** provides a complete detail of baseline characteristics.

**Table 1:** Baseline characteristics of training set and validation set in patients.

Characteristic	Training set (n=355)	Validation set (n=151)
Sex, n (%)		
Male	212 (59.7%)	85 (56.3%)
Female	143 (40.3%)	66 (43.7%)
Age (y), mean (SD)	57.24 (13.47)	58.27 (13.83)
Smoking		
Yes	146 (41.13%)	74 (49.01%)
No	209 (58.87%)	77 (50.99%)
Hypertension		
Yes	112 (31.55%)	51 (33.77%)
No	243 (68.45%)	100 (66.23%)
Diabetes		
Yes	111 (31.27%)	49 (32.45%)
No	244 (68.73%)	102 (67.55%)
Body Mass Index, mean (SD)	21.65 (2.74)	21.76 (3.18)
Tumor Length, mean (SD)	4.14 (1.84)	4.07 (1.77)
Tumor Location, n (%)		
Body	30 (8.5%)	11 (7.3%)
Head	239 (67.3%)	105 (69.5%)
Neck	14 (3.9%)	11 (7.3%)
Tail	72 (20.3%)	24 (15.9%)
T, n (%)		
T1	48 (13.5%)	23 (15.2%)
T2	164 (46.2%)	66 (43.7%)
T3	143 (40.3%)	62 (41.1%)
N, n (%)		
N0	153 (43.1%)	78 (51.7%)
N1	202 (56.9%)	73 (48.3%)
TNM Stage, n (%)		
T1N0M0	31 (8.7%)	14 (9.3%)
T1N1M0	17 (4.8%)	9 (6.0%)
T2N0M0	88 (24.8%)	45 (29.8%)
T2N1M0	76 (21.4%)	21 (13.9%)
T3N0M0	34 (9.6%)	19 (12.6%)
T3N1M0	109 (30.7%)	43 (28.5%)
Stage, n (%)		
IA	31 (8.7%)	14 (9.3%)
IB	88 (24.8%)	45 (29.8%)
IIA	34 (9.6%)	19 (12.6%)
IIB	202 (56.9%)	73 (48.3%)
Pathological Grading, n (%)		
Well-differentiated (G1)	48 (13.5%)	17 (11.3%)
Moderately differentiated (G2)	244 (68.7%)	110 (72.8%)
Poorly differentiated (G3)	63 (17.7%)	24 (15.9%)
AFP (ng/mL), mean (SD)	3.46 (1.59)	3.71 (1.65)
CEA (ng/mL), mean (SD)	6.81 (10.29)	5.56 (7.59)
CA19-9 (U/mL), mean (SD)	358.96 (397.97)	331.43 (388.98)
CA125 (U/mL), mean (SD)	33.63 (47.74)	36.55 (66.53)
Total Bilirubin (μmol/L), mean (SD)	106.50 (43.74)	102.62 (40.22)
Direct Bilirubin (μmol/L), mean (SD)	87.51 (38.47)	86.01 (36.93)
Indirect Bilirubin (μmol/L), mean (SD)	18.99 (8.19)	16.61 (7.50)
Amylase (U/L), mean (SD)	100.39 (69.91)	146.07 (49.96)
Lipase (U/L), mean (SD)	119.93 (76.11)	188.66 (70.03)
Fasting Blood Glucose (mmol/L), mean (SD)	6.89 (1.91)	6.75 (1.88)
Triglyceride (mmol/L), mean (SD)	2.22 (1.07)	2.20 (1.11)
TyG index, mean (SD)	9.28 (0.67)	9.25 (0.71)
Total Protein (g/L), mean (SD)	71.73 (10.55)	70.25 (10.82)
Albumin (g/L), mean (SD)	40.10 (8.66)	39.21 (9.64)
White Blood Cell (×10^9/L), mean (SD)	7.77 (3.47)	7.8 (3.15)
Neutrophil (×10^9/L), mean (SD)	4.78 (2.36)	4.78 (2.08)
Lymphocyte (×10^9/L), mean (SD)	2.14 (1.04)	2.14 (0.99)
NLR, mean (SD)	2.42 (0.84)	2.45 (0.78)
Red Blood Cell (×10^12/L), mean (SD)	4.56 (0.51)	4.54 (0.71)
Hemoglobin (g/L), mean (SD)	136.41 (15.73)	134.94 (18.26)
Platelet (×10^9/L), mean (SD)	217.53 (39.66)	201.32 (55.24)

SD, Standard Deviation; AFP, Alpha-Fetoprotein; CEA, Carcinoembryonic Antigen; CA19-9, Carbohydrate Antigen 19-9; CA125, Carbohydrate Antigen 125; TyG index, Triglyceride Glucose Index; NLR, Neutrophil to Lymphocyte Ratio.

### Univariate analysis

We next screened candidate predictors using univariate Cox regression. Several clinical variables were associated with poorer survival. Higher BMI, hypertension, elevated indirect bilirubin, and elevated lipase predicted increased mortality, while total protein, albumin, and hemoglobin showed protective associations. Among metabolic markers, fasting glucose, triglyceride, and the TyG index all predicted poorer survival, with the TyG index generating the highest hazard ratio. For pathological factors, longer tumor length, more advanced TNM stage, and higher pathological grade each correlated with shorter survival, consistent with established prognostic knowledge. All univariate results are presented in **Table 2**.

**Table 2:** Univariate cox regression analyses.

Variable	Hazard Ratio	95% confidence interval	*P*
Sex	1.010	0.812-1.257	0.929
Smoking	1.213	0.98-1.506	**0.080**
Hypertension	1.450	1.144-1.838	**0.002**
Diabetes	0.913	0.726-1.147	0.433
Age	1.004	0.996-1.011	0.363
Body Mass Index	1.063	1.022-1.106	**0.002**
AFP	0.983	0.921-1.049	0.600
CEA	0.999	0.990-1.009	0.844
CA19-9	1.000	0.999-1.000	0.128
CA125	1.001	0.999-1.004	0.387
Total Bilirubin	1.000	0.999-1.000	0.282
Direct Bilirubin	1.001	0.999-1.001	0.235
Indirect Bilirubin	1.005	1.001-1.010	**0.030**
Amylase	0.999	0.999-1.000	0.154
Lipase	1.000	0.999-1.000	**0.043**
Total Protein	0.988	0.978-0.999	**0.024**
Albumin	0.978	0.966-0.990	**<0.001**
White Blood Cell	0.979	0.950-1.009	0.169
Neutrophil	0.979	0.936-1.024	0.349
Lymphocyte	0.931	0.839-1.034	0.183
NLR	1.073	0.943-1.220	0.286
Red Blood Cell	0.887	0.707-1.113	0.301
Hemoglobin	0.990	0.983-0.998	**0.010**
Platelet	1.000	0.997-1.003	0.735
Fasting Blood Glucose	1.074	1.015-1.136	**0.014**
Triglyceride	1.243	1.123-1.376	**<0.001**
TyG Index	1.433	1.212-1.695	**<0.001**
Tumor Location	1.090	0.739-0.634	0.665
Tumor Length	1.091	1.036-1.150	**0.001**
TNM Stage	2.627	1.363-1.502	**0.004**
Pathological Grading	1.652	1.183-1.137	**0.003**

AFP, Alpha-Fetoprotein; CEA, Carcinoembryonic Antigen, CA19-9, Carbohydrate Antigen 19-9; CA125, Carbohydrate Antigen 125; NLR, Neutrophil to Lymphocyte Ratio; TyG index, Triglyceride Glucose Index.

Bold values indicate p < 0.05.

### Multivariate analysis

Variables significant at p < 0.10 in univariate analysis were entered into multivariable Cox models. In the final clinical model, smoking, hypertension, BMI, indirect bilirubin, albumin, hemoglobin, and the TyG index remain independent predictors. The TyG index again carried the largest hazard ratio. Lipase, total protein, fasting glucose, and triglyceride lost significance after adjustment. In the pathological model, TNM stage and pathological grading independently predicted OS. Compared with T1N0M0, T3N0M0 conferred the highest risk (HR about 5.7), followed by T3N1M0 (HR about 4.0). Tumor length did not remain significant. The combined model integrated all predictors from the clinical and pathological models. Every variable retained its significance, and risk estimates were consistent with those from the individual models. **Table 3** displays the results from multivariate analysis for three models.

**Table 3:** Multivariate cox regression: independent predictors of overall survival.

Model/variable	Hazard ratio	95% confidence interval	*P*
Clinical Model			
Smoking			
Yes	1.322	1.061-1.649	**0.013**
No	ref	ref	/
Hypertension			
Yes	1.394	1.095-1.773	**0.007**
No	ref	ref	/
Body Mass Index	1.074	1.032-1.119	**<0.001**
Indirect Bilirubin	1.006	1.002-1.011	**0.009**
Lipase	0.999	0.999-1.000	0.118
Total Protein	1.019	0.993-1.045	0.159
Albumin	0.979	0.967-0.991	**<0.001**
Hemoglobin	0.990	0.983-0.997	**0.007**
Fasting Blood Glucose	0.945	0.803-1.112	0.493
Triglyceride	0.968	0.628-1.493	0.882
TyG Index	1.433	1.207-1.703	**<0.001**
Patdological Model			
Tumor Lengtd	0.926	0.8071-1.0615	0.268
TNM Stage			
T1N0M0	ref	ref	/
T1N1M0	2.395	1.23-4.675	**0.0105**
T2N0M0	2.610	1.482-4.591	**<0.001**
T2N1M0	3.041	1.711-5.404	**<0.001**
T3N0M0	5.747	2.710-12.185	**<0.001**
T3N1M0	4.185	1.801-9.725	**<0.001**
Patdological Grading			
Well-differentiated (G1)	ref	ref	/
Moderately differentiated (G2)	1.558	1.098-2.211	**0.013**
Poorly differentiated (G3)	1.407	0.940-2.107	**0.047**
Combined Model			
Smoking			
Yes	1.305	1.041-1.636	**0.021**
No	ref	ref	/
Hypertension			
Yes	1.442	1.124-1.849	**0.004**
No	ref	ref	/
Body Mass Index	1.077	1.034-1.122	**<0.001**
Indirect Bilirubin	1.005	0.999-1.010	**0.048**
Albumin	0.980	0.968-0.992	**0.001**
Hemoglobin	0.992	0.984-0.999	**0.031**
TyG Index	1.496	1.246-1.797	**<0.001**
TNM Stage			
T1N0M0	ref	ref	/
T1N1M0	2.145	1.087-4.231	**0.028**
T2N0M0	2.620	1.473-4.662	**0.001**
T2N1M0	3.171	1.771-5.676	**<0.001**
T3N0M0	5.702	2.658-12.235	**<0.001**
T3N1M0	4.334	1.864-10.079	**<0.001**
Patdological Grading			
Well-differentiated (G1)	ref	ref	/
Moderately differentiated (G2)	1.619	1.134-2.312	**0.008**
Poorly differentiated (G3)	1.465	0.968-2.217	**0.048**

Variables with *P* < 0.05 are considered independent prognostic factors. TyG index, Triglyceride Glucose Index.

Bold values indicate p < 0.05.

The proportional hazards assumption was assessed using Schoenfeld residual tests for all three models. The pathological model showed no evidence of PH violation (global P = 0.902). However, the clinical and combined models exhibited time-varying effects, primarily driven by BMI and TyG (**Supplementary Table 1**). For the combined model, we included time-dependent interaction terms for these two variables using a logarithmic time transformation; both interaction terms were statistically significant (P = 0.004 and P < 0.001, respectively). Stratified analysis by early (≤12 months) versus late (>12 months) follow-up further revealed that the prognostic effect of TyG was substantially stronger in the early postoperative period (HR = 1.917) than in the later period (HR = 1.213), while the effect of BMI shifted from protective in the early period (HR = 0.866) to adverse in the late period (HR = 1.055). After incorporating these time-dependent terms into the combined model, all remaining variables satisfied the PH assumption.

### Model development

**Figure 2:**
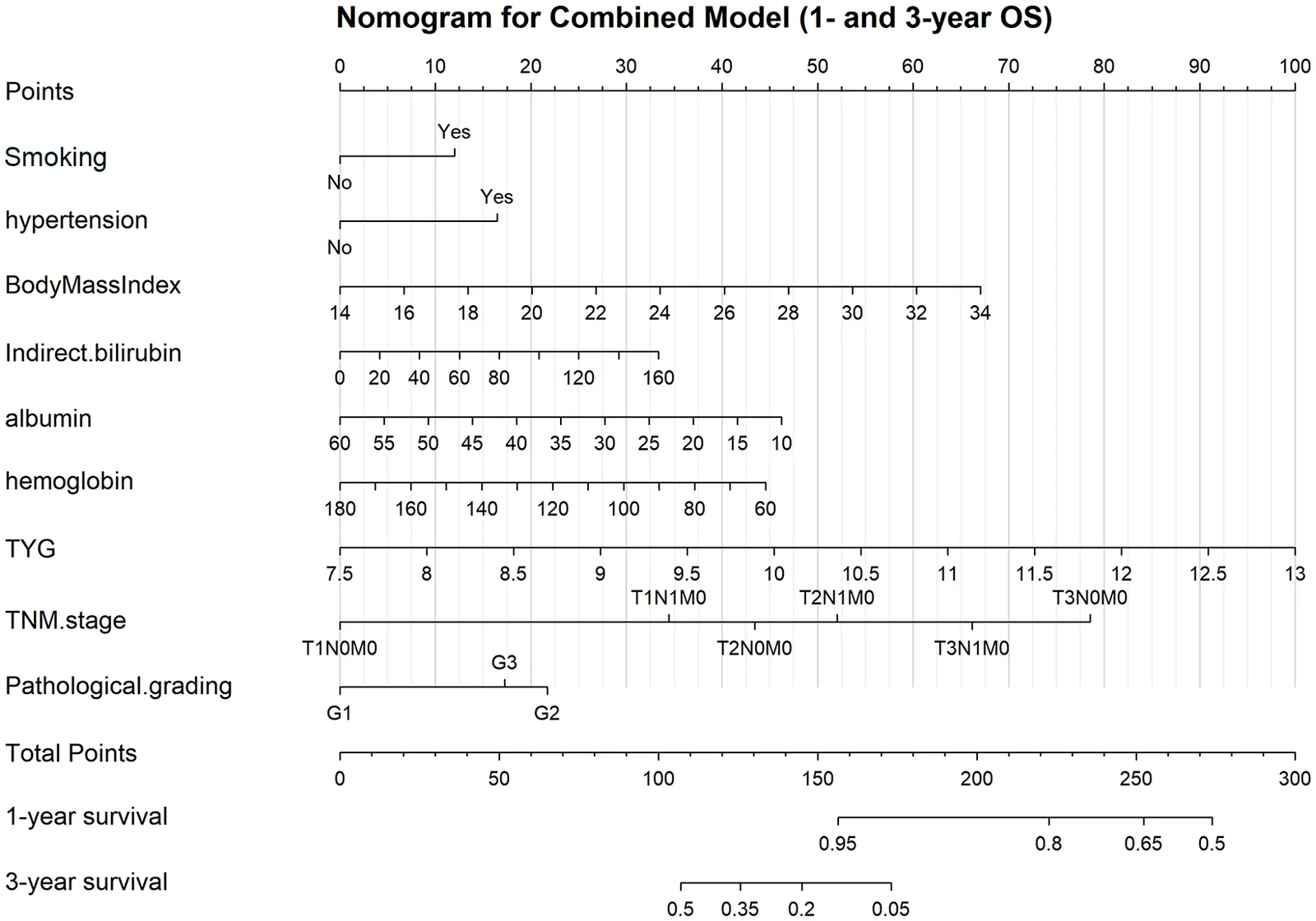
Nomogram for predicting 1-year and 3-year overall survival based on the combined model. To use the nomogram, locate each predictor variable on its axis, draw a vertical line to the points scale to obtain the points, sum the points, and then read the 1-year and 3-year survival probabilities from the total points axis. An example patient is shown with red dots.

We translated each multivariable model into a linear predictor for risk scoring. The clinical model incorporates seven variables; the pathological model relies solely on TNM stage and grading; the combined model pools both sets. **Table 4** lists the corresponding formulas. These linear predictors form the basis for individualized survival probability estimation. Additionally, we constructed a nomogram of the combined model to facilitate individualized prediction of 1-year and 3-year OS (**Figure 2**). The nomogram assigns points to each predictor according to its regression coefficient. The points can be summed for a given patient, and the corresponding survival probabilities can be read from the bottom scale.

**Table 4:** Linear predictor formulas for risk scores.

Model	Risk score formula
Clinical Model	0.2794 × Smoking + 0.3319 × Hypertension + 0.0717 × BMI + 0.0064 × Indirect Bilirubin – 0.0214 × Albumin – 0.0100 × Hemoglobin + 0.3599 × TyG Index
Pathological Model	0.8734 × T1N1M0 + 0.9586 × T2N0M0 + 1.1123 × T2N1M0 + 1.7486 × T3N0M0 + 1.4315 × T3N1M0 + 0.4434 × G2 + 0.3416 × G3
Combined Model	0.2664 × Smoking + 0.3657 × Hypertension + 0.0743 × BMI + 0.0046 × Indirect Bilirubin – 0.0205 × Albumin – 0.0082 × Hemoglobin + 0.4029 × TyG index + 0.7631 × T1N1M0 + 0.9632 × T2N0M0 + 1.1539 × T2N1M0 + 1.7409 × T3N0M0 + 1.4665 × T3N1M0 + 0.4820 × G2 + 0.3821 × G3

For categorical variables, Smoking and Hypertension take 1 for “Yes” and 0 for “No”. For TNM stage and pathological grading, each specific subgroup takes 1 if the patient belongs to that category and 0 otherwise. The reference categories are non-smokers, no hypertension, TNM stage T1N0M0, and well-differentiated (G1). All continuous variables (BMI, indirect bilirubin, albumin, hemoglobin, and TyG index) enter the formula with their original measured values. The linear predictor equals the sum of the products shown above.

BMI, Body Mass Index; TyG index, Triglyceride Glucose Index.

We provide a prediction example using a randomly selected patient from the validation set. This patient was chosen at random without any preselection based on outcomes or risk profile. With a linear predictor of 1.866, this patient’s risk score fell at the 49.7th percentile of the validation set distribution (median = 1.8661), confirming that this case is representative of a typical intermediate-risk patient rather than an extreme outlier. This patient had no smoking history, a diagnosis of hypertension, a body mass index of 21.36 kg/m², an indirect bilirubin level of 17.9 μmol/L, serum albumin of 39 g/L, hemoglobin of 118.3 g/L, a TyG index of 8.98, TNM stage T2N0M0, and moderately differentiated tumor (G2). The calculated linear predictor for this patient was 1.866. The nomogram predicted a 1-year survival probability of 0.46 and a 3-year survival probability < 0.05, indicating a very poor long-term prognosis.

### Model performance

Calibration was assessed at 12 and 36 months using ten equal-sized groups. Full results appear in **Supplementary Table 2**. At 12 months, the pathological model calibrated best: R² reached 0.405, the intercept sat near zero, and the slope stayed close to 1 (1.033). At 36 months, however, this advantage faded: the slope fell to 0.473 and R² dropped to 0.290. The clinical and combined models showed weaker calibration overall. The clinical model’s slope deviated clearly from the ideal line at both time points. The combined model had a slope near zero at 12 months, improving somewhat to 0.407 at 36 months with an R² of 0.211. Calibration curves for 12 and 36 months are shown in **Supplementary Figure 1**.

Discrimination metrics are summarized in **Table 5**. The combined model outperformed the other two. In the validation set, its C-index reached 0.721 for both time points, with time-dependent AUCs of 0.821 (12 months) and 0.998 (36 months). This 36-month AUC estimate is almost certainly inflated by the small number of patients remaining at risk at that time point and should be interpreted with caution, not as evidence of near-perfect discrimination. Sensitivity exceeded 0.78 throughout. The clinical model followed, with C-indices of 0.684 and AUCs of 0.801 and 0.947. The pathological model lagged behind-validation C-index 0.618, 12-month AUC only 0.587, though its 36-month AUC was 0.993. Training-set results tracked those in the validation set.

**Table 5:** Discrimination performance of the three models in training and validation sets.

Dataset/Model	Time	C-index	C-index 95% CI	AUC	Accuracy	Sensitivity	Specificity	PPV	NPV
Training set									
Clinical	1-year	0.655	0.606-0.705	0.790	0.797	0.696	0.812	0.356	0.947
Clinical	3-year	0.655	0.606-0.705	0.740	0.947	0.952	0.500	0.994	0.111
Pathological	1-year	0.582	0.531-0.634	0.617	0.732	0.413	0.780	0.218	0.899
Pathological	3-year	0.582	0.531-0.634	0.953	0.914	0.913	1.000	1.000	0.121
Combined	1-year	0.681	0.633-0.730	0.807	0.713	0.761	0.706	0.278	0.952
Combined	3-year	0.681	0.633-0.730	0.981	0.970	0.970	1.000	1.000	0.286
Validation set									
Clinical	1-year	0.684	0.609-0.758	0.801	0.775	0.739	0.781	0.378	0.943
Clinical	3-year	0.684	0.609-0.758	0.947	0.929	0.929	1.000	1.000	0.091
Pathological	1-year	0.618	0.540-0.696	0.587	0.689	0.435	0.734	0.227	0.879
Pathological	3-year	0.618	0.540-0.696	0.993	0.922	0.921	1.000	1.000	0.083
Combined	1-year	0.721	0.650-0.793	0.821	0.676	0.783	0.656	0.290	0.944
Combined	3-year	0.721	0.650-0.793	0.998	0.950	0.950	1.000	1.000	0.125

CI, confidence interval; AUC, area under the time-dependent receiver operating characteristic curve; PPV, positive predictive value; NPV, negative predictive value.

### Prognostic and decision analyses

**Figure 3:**
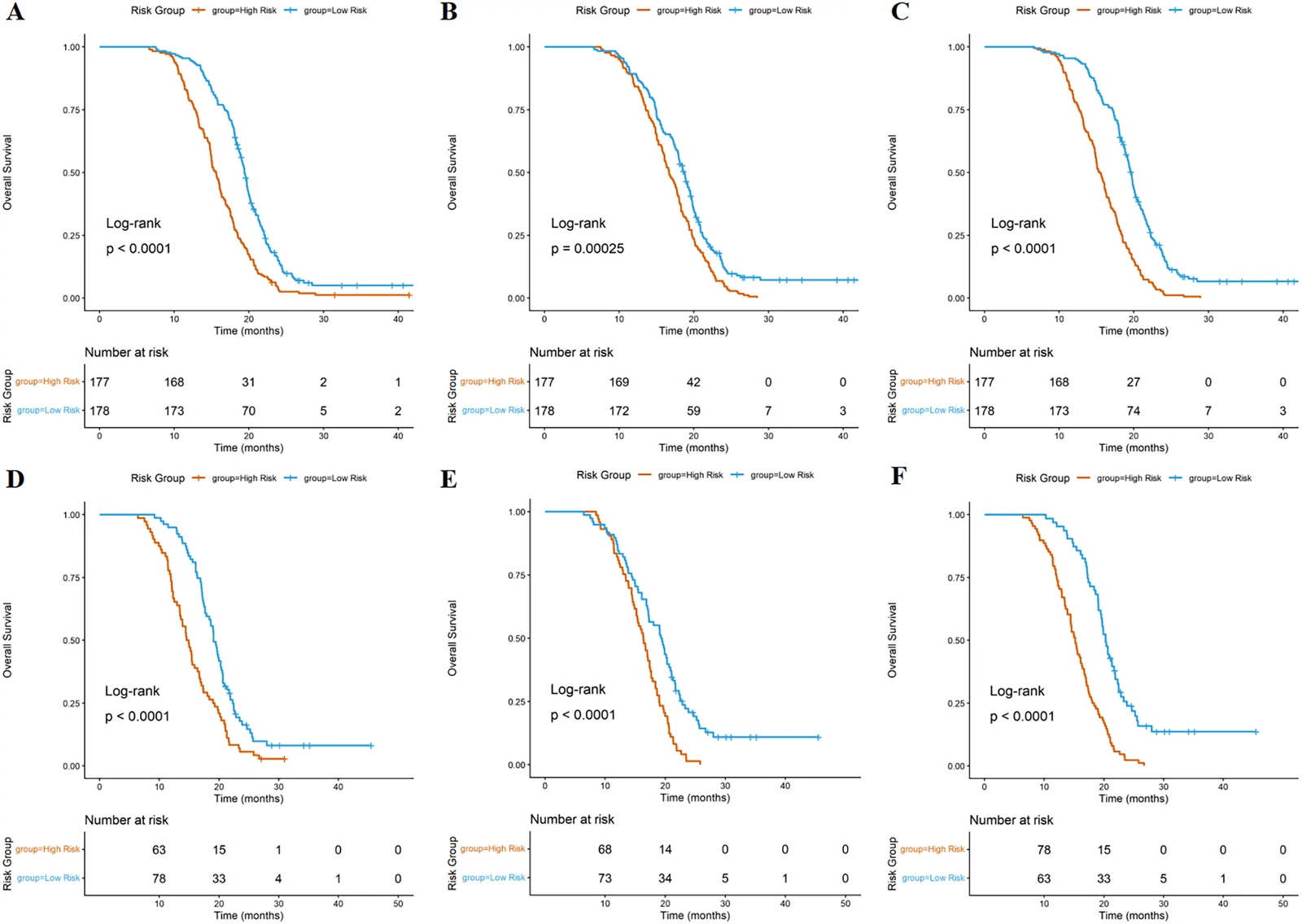
Kaplan-Meier curves for overall survival according to risk group defined by each model. **(A)** Clinical model in the training set. **(B)** Pathological model in the training set. **(C)** Combined model in the training set. **(D)** Clinical model in the validation set. **(E)** Pathological model in the validation set. **(F)** Combined model in the validation set. We determined the high-risk and low-risk groups using the median risk score of each model in the training set. The tables below each curve show the number at risk at 0, 10, 20, 30 and 40 months for the high-risk and low-risk groups. The shaded areas represent 95% confidence intervals. P values from log-rank tests were all below 0.05.

We used the median risk score of each model in the training set to classify patients into high-risk and low-risk groups. **Figures 3A-C** present the Kaplan-Meier curves for the clinical, pathological, and combined models in the training set, respectively. **Figures 3D-E** show the corresponding curves for the validation set. The log-rank test confirmed significant differences between the two risk groups for all three models in both datasets (all P < 0.05). Reflecting the generally poor prognosis of pancreatic ductal adenocarcinoma, the 3-year survival rate remained below 0.25 for both high-risk and low-risk groups across all models. Nevertheless, the combined model produced the most pronounced separation between the two groups, with a notably wider gap in survival curves compared to the clinical or pathological models. This suggests improved relative risk discrimination, even though absolute survival was poor.

**Figure 4:**
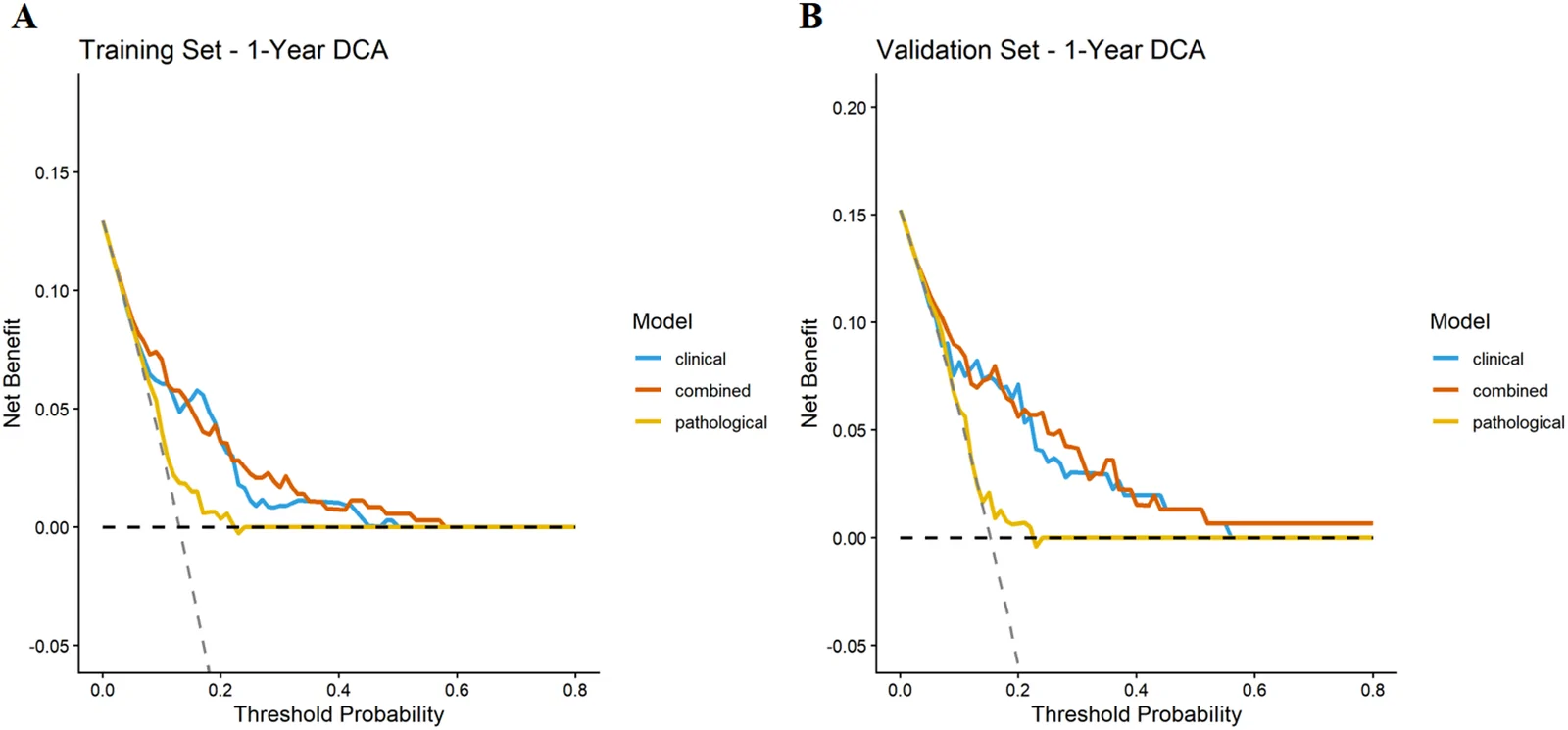
Decision curve analysis for 1-year overall survival. **(A)** Training set. **(B)** Validation set. The y-axis shows net benefit, and the x-axis shows threshold probability. Lines represent the clinical model (blue), pathological model (yellow), and combined model (red). The gray dashed line indicates the treat-all strategy, and the black dashed line indicates the treat-none strategy.

Decision curve analysis focused on 1-year predictions, as the high mortality of PDAC limits the relevance of longer horizons. **Figure 4** shows the net benefit curves (A: training; B: validation). The combined model yielded the highest net benefit across most threshold probabilities, especially in the lower range where intervention is more likely. The clinical model also delivered positive net benefit, trailing the combined model only slightly. The pathological model fell below both. Net benefit differences between models were largest at low thresholds and narrowed as thresholds increased, with all curves eventually converging toward zero. These findings support the combined model’s greater clinical utility for guiding early postoperative decisions.

### Risk score distribution

**Figure 5:**
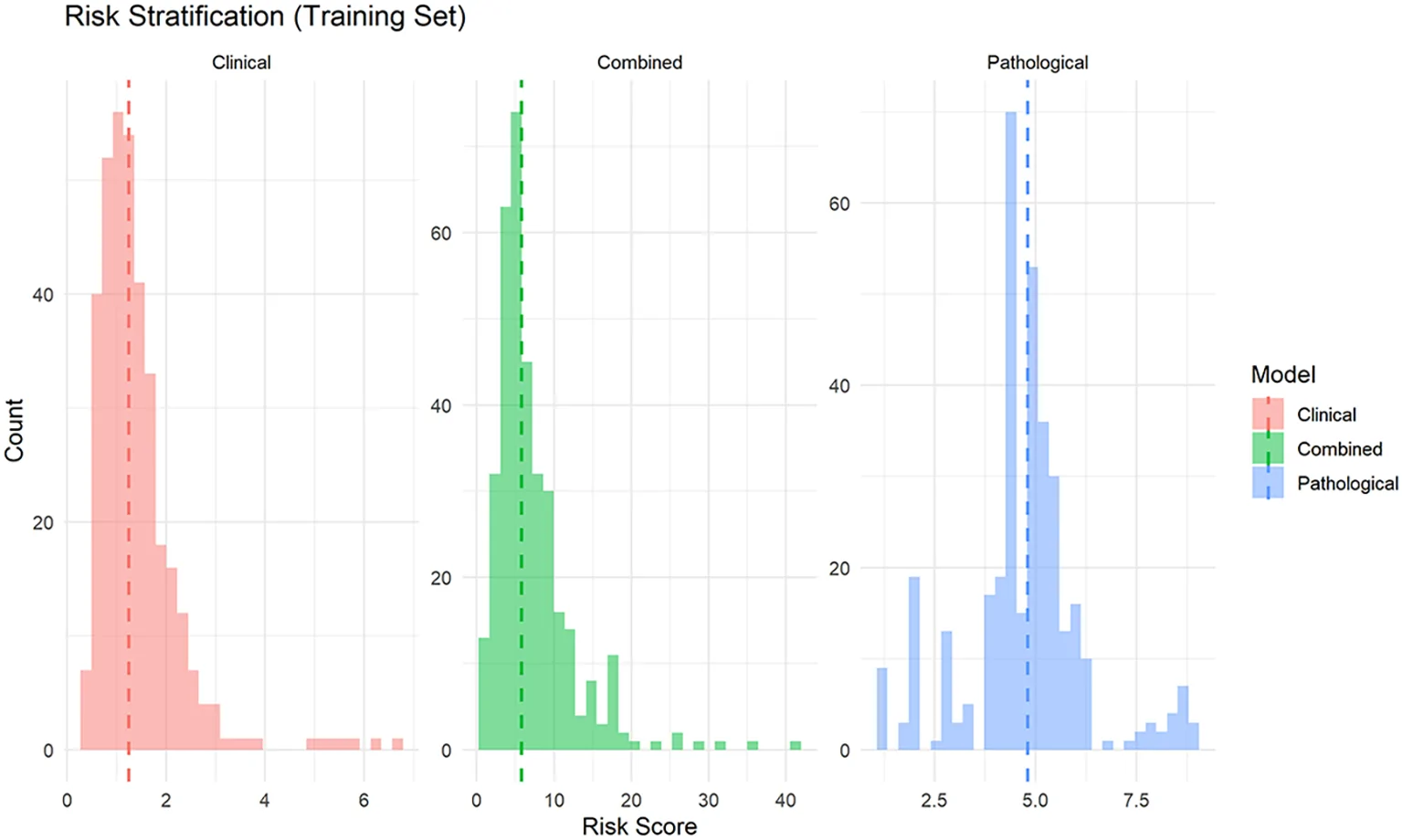
Risk score distribution in the training set for the three models. Risk score distribution in the training set for the three models. Histograms show the spread of risk scores for the clinical, pathological, and combined models. Vertical dashed lines indicate the median risk score of each model, used as the cutoff to define high-risk and low-risk groups. The x-axis represents the risk score, and the y-axis shows the count of patients.

Risk score histograms for the training set, with median cutoffs marked, are provided in **Figure 5**. All three models generated a wide score spread, indicating substantial inter-patient heterogeneity. The clinical model’s distribution showed a long right tail, while the pathological and combined models appeared more symmetric. For the combined model, we found the median to be approximately 6.5, with scores spanning from near 0 to above 40. Visual separation of high- and low-risk groups was clear in all cases, supporting the median as a reasonable threshold.

### Subgroup analysis

**Figure 6:**
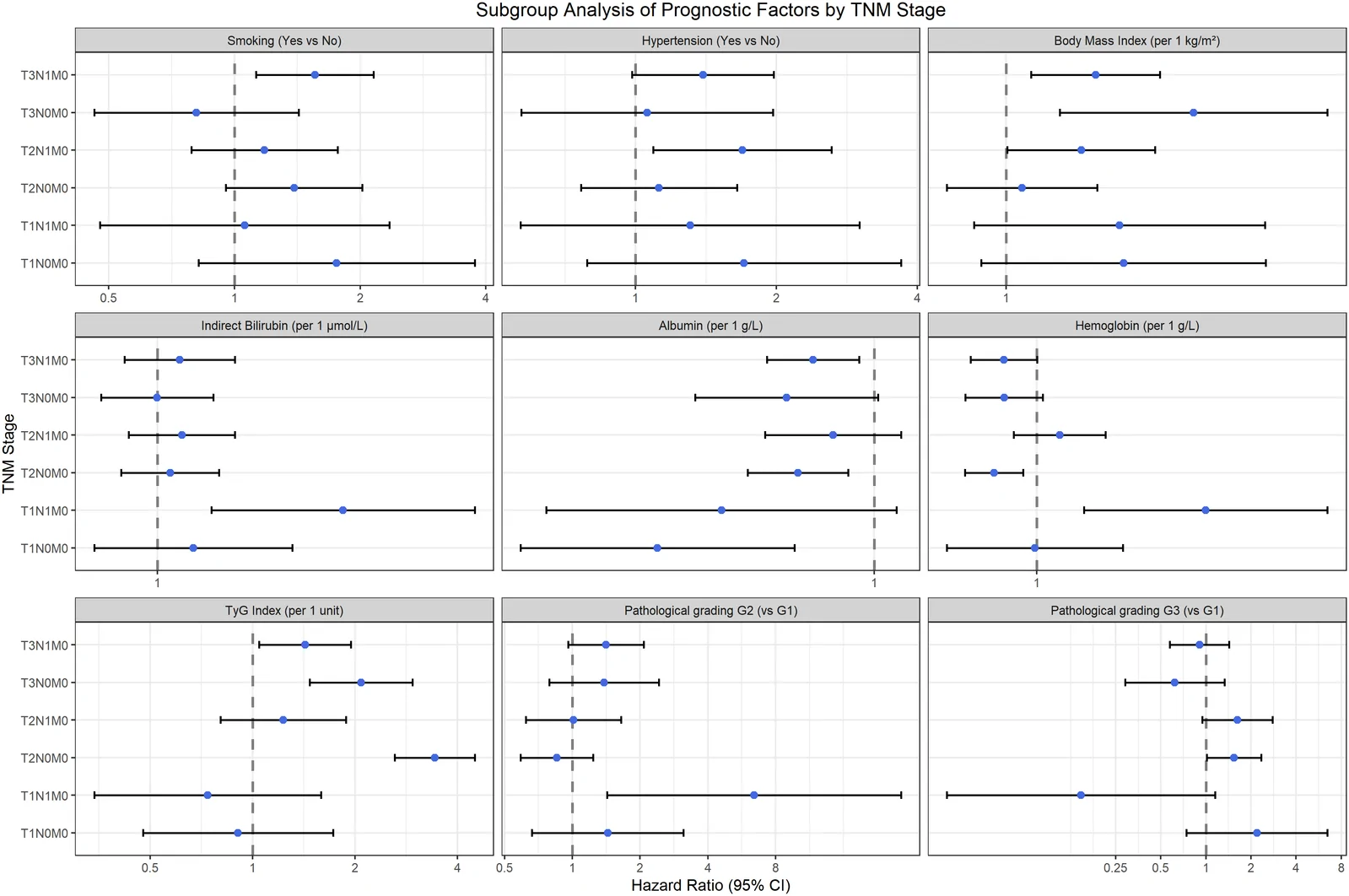
Forest plot of subgroup analysis by TNM stage. The plot shows hazard ratios (squares) with 95% confidence intervals (horizontal lines) for the association of each predictor with OS within each TNM subgroup. The vertical dashed line indicates a hazard ratio of 1. Symbols to the right of the line indicate increased risk, and those to the left indicate decreased risk.

We next examined whether prognostic associations varied by TNM stage. Hazard ratios for each predictor within six TNM subgroups are listed in **Supplementary Table 3**, and the forest plot appears in **Figure 6**. The TyG index exhibited the most prominent stage-dependent effect. In T2N0M0 and T3N0M0 disease, higher TyG predicted sharply worse survival (HR 3.43 and 2.09, both P < 0.001); in T3N1M0, the effect attenuated but remained significant (HR 1.43, P = 0.025). In earlier stages, no association emerged. Albumin consistently lowered risk across most subgroups, with HRs ranging from 0.93 (T1N0M0) to 0.98 (T3N1M0). BMI predicted worse survival predominantly in advanced disease: T2N1M0 (HR 1.07, P = 0.047), T3N0M0 (HR 1.19, P = 0.006), T3N1M0 (HR 1.09, P = 0.007).

Several other associations were confined to single subgroups. Indirect bilirubin raised risk in T1N1M0 (HR 1.03, P = 0.006). Hemoglobin showed protective effects in T1N1M0 and T2N0M0. Hypertension increased risk in T2N1M0 (HR 1.69, P = 0.019), smoking in T3N1M0 (HR 1.56, P = 0.008). For grading, G2 tumors carried substantially higher risk in T1N1M0 (HR 6.41, P = 0.015), and G3 in T2N0M0 (HR 1.54, P = 0.042). Taken together, the prognostic weight of metabolic and nutritional variables differed considerably across TNM stages, reinforcing the value of stage-stratified assessment. These subgroup analyses were exploratory in nature, and no adjustment for multiple comparisons was applied; findings should be interpreted with appropriate caution.

## Discussion

This retrospective analysis developed three prognostic models for resected PDAC using only routine data. Adding metabolic markers to standard pathology improved discrimination and net benefit. Systemic metabolic dysregulation may actively shape postoperative outcomes, rather than merely accompanying the disease.

The links between metabolism and PDAC are well documented. Diabetes is a well-established risk factor for PDAC, and conversely, PDAC itself can cause new-onset diabetes (31–33). Insulin resistance contributes to pancreatic carcinogenesis partly through the PI3K-Akt-mTOR axis and insulin-like growth factor signaling (34–37). The TyG index, a practical surrogate for insulin resistance derived from fasting triglyceride and glucose, has been associated with worse survival in several cancer types (38). In patients with pancreatic cancer, metabolic and inflammatory parameters measured at diagnosis have also been shown to correlate with disease stage and overall prognosis, further supporting the clinical relevance of systemic metabolic assessment in this malignancy (39). Our findings extend this association to the postoperative setting: a higher preoperative TyG index independently predicted shorter OS. What caught our attention was the striking cancer stage-dependence. The TyG index was most strongly associated with survival in T2N0M0 and T3N0M0 disease. In node-positive subgroups, its influence diminished. One interpretation is that once extensive nodal spread is established, lymphovascular invasion or systemic inflammation overwhelm the risk contribution of insulin resistance. Smaller patient numbers in some strata may also explain part of the variability. Regardless, TyG appears informative before nodal involvement becomes extensive. This hypothesis needs testing in larger cohorts.

The time-dependent patterns of TyG and BMI merit additional consideration. The stronger TyG effect in the early postoperative period suggests that insulin resistance may be particularly influential during the phase of early recurrence and rapid progression, potentially reflecting its role in promoting tumor proliferation and metastatic spread. As the disease stabilizes or transitions to a chronic phase, other factors such as cachexia or treatment-related effects may dilute the prognostic signal. The observed shift of BMI from protective to adverse over time is intriguing; one possible explanation is that higher BMI provides a metabolic reserve that transiently buffers against early mortality, but in the long term, obesity-related inflammation and metabolic dysfunction have a net harmful effect. This temporal nuance may partially explain the previously described “obesity paradox” in pancreatic cancer and warrants further investigation in larger prospective cohorts.

Albumin and BMI showed similar stage-specific associations. Low albumin predicted worse survival, consistent with the documented impact of malnutrition and cachexia in PDAC (40–43). High BMI predicted worse outcomes in advanced stages, aligning with the harmful effects of obesity on cancer progression (44, 45). Chronic inflammation, altered adipokine secretion, and heightened insulin resistance promote cancer development (46–48). An obesity paradox—in which higher BMI appears protective in some advanced cancers—has been described in some clinical settings (49–51). However, we found no such paradox in our study. The adverse effect of obesity dominated once disease had progressed. One possible explanation is that obesity has a true harmful effect rather than a protective one, as any potential “metabolic reserve” advantage of higher BMI may be outweighed by obesity-driven tumor aggressiveness once the disease has progressed.

Several other variables retained independent prognostic value. Smoking remained significant in the combined model and was especially detrimental in T3N1M0, hinting at possible synergy with advanced nodal burden. This remains speculative. The detrimental effects of smoking on pancreatic cancer are well documented, including increased risk of developing PDAC, earlier recurrence, and worse survival (52, 53). Hypertension predicted worse survival overall and reached significance in T2N1M0. Chronic hypertension is linked to vascular damage and systemic inflammation, which may contribute to tumor progression (54, 55). However, the literature on hypertension and cancer survival is mixed, and confounding by antihypertensive drugs cannot be ruled out (56–58). Our results should be interpreted cautiously. Higher hemoglobin was associated with better prognosis, which aligns with the well-recognized negative impact of anemia on cancer outcomes (59, 60). Anemia in pancreatic cancer can result from malnutrition, chronic disease, or tumor-induced bleeding (61, 62). This effect persisted after adjusting for nutritional markers, suggesting it captures aspects of physiological reserve beyond nutrition. Indirect bilirubin showed a modest positive association with mortality. The finding was unexpected but the underlying mechanism is unclear. It could reflect hepatic dysfunction or subclinical hemolysis driven by chronic inflammation (63).

TNM stage and histological grade were, as anticipated, the dominant predictors. Hazard ratios climbed with stage, approaching sixfold for T3N0M0 disease. Higher grades were associated with elevated risk (64, 65). These pathological factors set the baseline for prognosis; our metabolic and clinical variables refine the stratification within each stage.

Our models have practical advantages. Many published nomograms rely on specialized imaging or molecular assays (66, 67). Those tools often rely on complex variables or are derived from specific patient populations. Ours require only preoperative laboratory tests and postoperative pathology, including the TyG index from a standard metabolic panel. Recent studies have also explored prognostic modeling in advanced cancer patients using simple, routinely available parameters such as SARC-F and nutritional markers, further demonstrating the broader applicability of clinical-based risk stratification tools across different cancer populations (68). Several previous studies have investigated the prognostic role of the TyG index in pancreatic cancer. A study reported that a high TyG index predicts poor prognosis, especially in late-stage patients (69). Another study found that a lower TyG index was associated with a higher risk of liver metastasis (18). Some researchers constructed a prognostic model for unresectable PDAC using 3D CT-derived body composition and TyG-BMI (17). These studies on TyG in pancreatic cancer have largely focused on advanced or unresectable stages. We focus on the postoperative window, where risk stratification informs decisions about adjuvant therapy and follow-up intensity. The separate validation set, though internal, adds rigor.

The 12-month calibration of the clinical and combined models deserves specific attention. Their near-zero calibration slopes indicate that predicted absolute survival probabilities were not well aligned with observed outcomes across the full risk spectrum. This is not entirely unexpected in early postoperative PDAC, where survival is shaped by a confluence of factors that are difficult to capture with routine preoperative data alone, including performance status, postoperative complications, receipt and regimen of adjuvant chemotherapy, and competing non-cancer events. The 12-month window also coincides with the peak period of early recurrence, when biological heterogeneity may exert its strongest influence and temporarily overwhelm the prognostic signal from static metabolic markers. Inspection of the calibration curves further reveals that this miscalibration is not uniform across the risk spectrum. For the combined model, predictions in the low-risk range overestimate observed survival, while in the high-risk range the model underestimated it. This result suggests that the model may perform best for patients in the intermediate risk strata, and that extreme predictions at either end of the spectrum should be interpreted with particular caution. Despite this miscalibration in absolute terms, the model’s C-index and decision curve analysis still support its value for relative risk ordering, that is, distinguishing higher-risk from lower-risk patients, which is the more clinically actionable function when deciding which patients warrant closer surveillance or more aggressive adjuvant regimens. Clinicians should therefore interpret the numeric survival probabilities from the nomogram as directional risk indicators rather than precise point estimates.

How the risk groups separated over time in the Kaplan-Meier curves also warrants comment. The high-risk and low-risk groups diverged early, within the first 12 months after surgery, but the survival gap between them narrowed progressively beyond 24 months. This result reflects the uniformly poor long-term prognosis of resected PDAC, where most patients eventually succumb to recurrence regardless of their initial risk stratification. Importantly, this observation suggests that the model’s discriminatory power is driven primarily by early postoperative events—the period most relevant for decisions regarding adjuvant therapy intensity and early surveillance strategies—rather than by sustained long-term risk separation. Clinicians should therefore view the model as most informative for short to intermediate-term decision-making, particularly within the first 2 years after resection, and interpret the 3-year predictions with appropriate caution given the convergence of survival curves at later time points.

Several limitations should be acknowledged. The single-center retrospective design inevitably introduces potential selection bias, and external validation in independent cohorts is therefore urgently needed before this model can be adopted in routine clinical practice. The internal training-validation split used in this study, while useful for initial performance assessment, does not replace the rigorous testing required in external settings with different patient populations, treatment protocols, and laboratory standards. Beyond this, several unmeasured factors may cause residual confounding; for instance, adjuvant chemotherapy data were not available in this retrospective cohort, so their potential impact on overall survival could not be assessed. Furthermore, the TyG index was derived from a single preoperative measurement of fasting triglyceride and glucose, both of which can be influenced by short-term fluctuations related to diet, medications, or acute illness. A single measurement may therefore not fully capture a patient’s longitudinal metabolic state, and future studies incorporating repeated measurements or alternative obesity-related metrics such as TyG-BMI or TyG-waist circumference could provide more stable estimates of metabolic risk. The suboptimal 1-year calibration further suggests that PDAC biology is more complex than our routine variables can capture, reinforcing that these models are best viewed as decision-support tools rather than precise prognostic instruments. Finally, as noted above, the near-perfect 3-year AUC of 0.998 is almost certainly an overestimate. The small number of patients surviving to 36 months makes this estimate statistically unstable and highly sensitive to individual outcome events. Moreover, because our models were developed using variable selection on the training set, the performance metrics are likely optimistic; with a smaller effective sample size at the 3-year time point, the gap between apparent performance and true performance may be particularly pronounced. Internal validation through data splitting provides some reassurance, but it does not eliminate the risk of overfitting. This AUC should therefore be interpreted as an upper-bound estimate rather than a definitive measure of long-term predictive accuracy. In addition, the use of backward stepwise selection for variable identification, while practical for reducing model complexity, carries a risk of overfitting to the training data and may yield coefficients that are overly optimistic. The selected variable set would therefore benefit from validation in external cohorts before being adopted more widely in clinical practice.

## Conclusion

We built three prognostic models using routine preoperative and pathological data from 506 patients who underwent curative resection for PDAC. Integrating metabolic indicators, especially the TyG index, with TNM stage and grade improved predictive performance and yielded the highest net benefit in decision curve analysis. Metabolic status plays a measurable role in postoperative survival. The models remain decision-support tools and require external validation before wider adoption.

## Data Availability

The original contributions presented in the study are included in the article/**Supplementary Material**. Further inquiries can be directed to the corresponding author.

## Supplementary material

Supplementary Figure 1 Calibration curves at 12 and 36 months for the clinical, pathological, and combined models in the validation set. **(A)** Clinical model at 12 months; **(B)** Pathological model at 12 months; **(C)** Combined model at 12 months; **(D)** Clinical model at 36 months; **(E)** Pathological model at 36 months; **(F)** Combined model at 36 months. The red dashed line represents ideal prediction (intercept =0, slope =1). Blue points show observed vs predicted survival probabilities with 95% bootstrap confidence intervals.

Supplementary Table 1 Proportional hazards assumption assessment for the three Cox models.

Supplementary Table 2 Calibration performance of the three models at 12 and 36 months in the validation set.

Supplementary Table 3 Subgroup analysis of individual predictors by TNM stage.
