# Supplementary material for: From metabolic syndrome to survival: a prognostic model for PDAC incorporating insulin resistance markers and pathological variables
Source: Front Endocrinol (Lausanne). 2026 Sep 3;17:1942873. doi: 10.3389/fendo.2026.1942873 (PMC13581534; doi:10.3389/fendo.2026.1942873)
Supplement: Supplementary file 1 [file Table1.docx]

**Supplementary Table 1 Proportional hazards assumption assessment for the three Cox models**

| **Model/Variable** | **chisq** | **df** | ***P*** |
| --- | --- | --- | --- |
| **Clinical Model** |  |  |  |
| Somking | 0.0005 | 1 | 0.9830 |
| hypertension | 0.9930 | 1 | 0.3191 |
| BMI | 8.1800 | 1 | 0.0042 |
| Indirect bilirubin | 0.8660 | 1 | 0.3520 |
| albumin | 1.1500 | 1 | 0.2828 |
| hemoglobin | 0.0385 | 1 | 0.8444 |
| TyG | 20.0000 | 1 | ＜0.0001 |
| GLOBAL | 32.9000 | 7 | ＜0.0001 |
| **Pathological Model** |  |  |  |
| Tumor length | 0.0352 | 1 | 0.8518 |
| TNM stage | 2.6637 | 5 | 0.7516 |
| Pathological grading | 0.1736 | 2 | 0.9169 |
| GLOBAL | 3.4640 | 8 | 0.9019 |
| **Combined Model** |  |  |  |
| Somking | ＜0.0001 | 1 | 0.9985 |
| hypertension | 0.5420 | 1 | 0.4614 |
| BMI | 9.1700 | 1 | 0.0025 |
| BMI × log(time)* | NA | NA | NA |
| Indirect bilirubin | 1.4000 | 1 | 0.2367 |
| albumin | 0.377 | 1 | 0.5391 |
| hemoglobin | ＜0.0001 | 1 | 0.9748 |
| TyG | 20.2000 | 1 | ＜0.0001 |
| TyG × log(time)* | NA | NA | NA |
| Tumor length | 0.0495 | 1 | 0.8239 |
| TNM stage | 0.6830 | 5 | 0.9839 |
| Pathological grading | 0.5920 | 2 | 0.7438 |
| GLOBAL | 33.6000 | 15 | 0.0039 |

**Note：**For the combined model, BMI and TyG violated the PH assumption (P = 0.0025 and P = 7.1×10⁻⁶, respectively) and were addressed by including time‑dependent interaction terms using a logarithmic time transformation. Both interaction terms were statistically significant (BMI × log(time): P = 0.004; TyG × log(time): P < 0.001), confirming the appropriateness of this adjustment. The global Schoenfeld test is not applicable for models with time‑dependent terms.
