# Supplementary material for: From metabolic syndrome to survival: a prognostic model for PDAC incorporating insulin resistance markers and pathological variables
Source: Front Endocrinol (Lausanne). 2026 Sep 3;17:1942873. doi: 10.3389/fendo.2026.1942873 (PMC13581534; doi:10.3389/fendo.2026.1942873)
Supplement: Supplementary file 2 [file Table2.docx]

**Supplementary Table 2 Calibration performance of the three models at 12 and 36 months in the validation set**

| **Model** | **Time months** | **R squared** | **Intercept** | **Slope** |
| --- | --- | --- | --- | --- |
| Clinical | 12 | 0.003 | 0.907 | -0.109 |
| Clinical | 36 | 0.126 | 0.080 | -0.479 |
| Pathological | 12 | 0.405 | -0.056 | 1.033 |
| Pathological | 36 | 0.290 | 0.045 | 0.473 |
| Combined | 12 | 0.000 | 0.836 | -0.026 |
| Combined | 36 | 0.211 | 0.055 | 0.407 |

**Note:** Calibration was assessed using 10 equally sized groups based on predicted survival probabilities.
