# Supplementary material for: From metabolic syndrome to survival: a prognostic model for PDAC incorporating insulin resistance markers and pathological variables
Source: Front Endocrinol (Lausanne). 2026 Sep 3;17:1942873. doi: 10.3389/fendo.2026.1942873 (PMC13581534; doi:10.3389/fendo.2026.1942873)
Supplement: Supplementary file 3 [file Table3.docx]

**Supplementary Table 3 Subgroup analysis of individual predictors by TNM stage**

| **Variable** | **Subgroup** | **N** | **Events** | **HR** | **Lower CI** | **Upper CI** | **P** |
| --- | --- | --- | --- | --- | --- | --- | --- |
| Smoking | T1N0M0 | 45 | 27 | 1.757 | 0.821 | 3.760 | 0.147 |
| Smoking | T1N1M0 | 26 | 26 | 1.058 | 0.476 | 2.349 | 0.891 |
| Smoking | T2N0M0 | 133 | 119 | 1.390 | 0.954 | 2.025 | 0.087 |
| Smoking | T2N1M0 | 97 | 97 | 1.180 | 0.788 | 1.765 | 0.422 |
| Smoking | T3N0M0 | 53 | 53 | 0.811 | 0.461 | 1.425 | 0.466 |
| Smoking | T3N1M0 | 152 | 152 | 1.557 | 1.125 | 2.155 | **0.008** |
| Hypertension | T1N0M0 | 45 | 27 | 1.705 | 0.787 | 3.692 | 0.176 |
| Hypertension | T1N1M0 | 26 | 26 | 1.308 | 0.568 | 3.012 | 0.528 |
| Hypertension | T2N0M0 | 133 | 119 | 1.122 | 0.764 | 1.647 | 0.557 |
| Hypertension | T2N1M0 | 97 | 97 | 1.692 | 1.090 | 2.625 | **0.019** |
| Hypertension | T3N0M0 | 53 | 53 | 1.059 | 0.571 | 1.965 | 0.856 |
| Hypertension | T3N1M0 | 152 | 152 | 1.393 | 0.984 | 1.974 | 0.062 |
| Body Mass Index | T1N0M0 | 45 | 27 | 1.114 | 0.977 | 1.270 | 0.106 |
| Body Mass Index | T1N1M0 | 26 | 26 | 1.110 | 0.971 | 1.269 | 0.127 |
| Body Mass Index | T2N0M0 | 133 | 119 | 1.015 | 0.947 | 1.087 | 0.680 |
| Body Mass Index | T2N1M0 | 97 | 97 | 1.071 | 1.001 | 1.147 | **0.047** |
| Body Mass Index | T3N0M0 | 53 | 53 | 1.188 | 1.050 | 1.344 | **0.006** |
| Body Mass Index | T3N1M0 | 152 | 152 | 1.086 | 1.023 | 1.152 | **0.007** |
| Indirect Bilirubin | T1N0M0 | 45 | 27 | 1.006 | 0.990 | 1.023 | 0.479 |
| Indirect Bilirubin | T1N1M0 | 26 | 26 | 1.031 | 1.009 | 1.054 | **0.006** |
| Indirect Bilirubin | T2N0M0 | 133 | 119 | 1.002 | 0.994 | 1.010 | 0.611 |
| Indirect Bilirubin | T2N1M0 | 97 | 97 | 1.004 | 0.995 | 1.013 | 0.369 |
| Indirect Bilirubin | T3N0M0 | 53 | 53 | 1.000 | 0.991 | 1.009 | 0.988 |
| Indirect Bilirubin | T3N1M0 | 152 | 152 | 1.004 | 0.995 | 1.013 | 0.431 |
| Albumin | T1N0M0 | 45 | 27 | 0.925 | 0.881 | 0.972 | **0.002** |
| Albumin | T1N1M0 | 26 | 26 | 0.947 | 0.889 | 1.008 | 0.088 |
| Albumin | T2N0M0 | 133 | 119 | 0.973 | 0.956 | 0.991 | **0.003** |
| Albumin | T2N1M0 | 97 | 97 | 0.985 | 0.962 | 1.010 | 0.235 |
| Albumin | T3N0M0 | 53 | 53 | 0.969 | 0.938 | 1.001 | **0.060** |
| Albumin | T3N1M0 | 152 | 152 | 0.978 | 0.962 | 0.995 | **0.009** |
| Hemoglobin | T1N0M0 | 45 | 27 | 0.999 | 0.969 | 1.031 | 0.969 |
| Hemoglobin | T1N1M0 | 26 | 26 | 1.062 | 1.017 | 1.108 | **0.007** |
| Hemoglobin | T2N0M0 | 133 | 119 | 0.985 | 0.975 | 0.995 | **0.004** |
| Hemoglobin | T2N1M0 | 97 | 97 | 1.008 | 0.992 | 1.025 | 0.327 |
| Hemoglobin | T3N0M0 | 53 | 53 | 0.989 | 0.975 | 1.002 | 0.098 |
| Hemoglobin | T3N1M0 | 152 | 152 | 0.988 | 0.977 | 1.000 | 0.052 |
| TyG Index | T1N0M0 | 45 | 27 | 0.907 | 0.477 | 1.725 | 0.766 |
| TyG Index | T1N1M0 | 26 | 26 | 0.738 | 0.342 | 1.589 | 0.437 |
| TyG Index | T2N0M0 | 133 | 119 | 3.430 | 2.615 | 4.498 | **0.000** |
| TyG Index | T2N1M0 | 97 | 97 | 1.231 | 0.804 | 1.884 | 0.339 |
| TyG Index | T3N0M0 | 53 | 53 | 2.085 | 1.473 | 2.952 | **0.000** |
| TyG Index | T3N1M0 | 152 | 152 | 1.427 | 1.045 | 1.948 | **0.025** |
| Pathological Grading-G2 | T1N0M0 | 45 | 27 | 1.436 | 0.662 | 3.115 | 0.360 |
| Pathological Grading-G2 | T1N1M0 | 26 | 26 | 6.414 | 1.426 | 28.853 | **0.015** |
| Pathological Grading-G2 | T2N0M0 | 133 | 119 | 0.854 | 0.589 | 1.238 | 0.405 |
| Pathological Grading-G2 | T2N1M0 | 97 | 97 | 1.011 | 0.622 | 1.644 | 0.965 |
| Pathological Grading-G2 | T3N0M0 | 53 | 53 | 1.384 | 0.790 | 2.425 | 0.256 |
| Pathological Grading-G2 | T3N1M0 | 152 | 152 | 1.410 | 0.957 | 2.077 | 0.083 |
| Pathological Grading-G3 | T1N0M0 | 45 | 27 | 2.187 | 0.739 | 6.476 | 0.158 |
| Pathological Grading-G3 | T1N1M0 | 26 | 26 | 0.147 | 0.019 | 1.151 | 0.068 |
| Pathological Grading-G3 | T2N0M0 | 133 | 119 | 1.542 | 1.016 | 2.341 | **0.042** |
| Pathological Grading-G3 | T2N1M0 | 97 | 97 | 1.620 | 0.942 | 2.786 | 0.081 |
| Pathological Grading-G3 | T3N0M0 | 53 | 53 | 0.619 | 0.288 | 1.330 | 0.219 |
| Pathological Grading-G3 | T3N1M0 | 152 | 152 | 0.906 | 0.575 | 1.428 | 0.670 |

**Note:** N and Events are derived from the combined dataset (training set + validation set, n = 506). We stratified patients by six TNM categories and performed univariate Cox regression for each predictor within each subgroup. Hazard ratios, 95% confidence intervals, and P values are reported. P values < 0.05 are shown in bold in the main manuscript.
